# A Novel Selective Strategy for Bioactive Limbal Stem Cells Primary Culture Using Deep Cryopreservation and IL-1β Precondition

**DOI:** 10.3390/cells15100880

**Published:** 2026-05-12

**Authors:** Yinglin Liu, Liling Xu, Yanmei Li, Cheng Lu, Zepei Fan, Jun Ling, Yingwei Wang, Zheng Wu

**Affiliations:** 1 Key Laboratory for Regenerative Medicine, Ministry of Education, Department of Developmental and Regenerative Biology, Jinan University, Guangzhou 510630, China; yinglinliu@jnu.edu.cn (Y.L.); 15820391659@163.com (L.X.); liyanmei010911@163.com (Y.L.); lucheng@jnu.edu.cn (C.L.); zepeifan_jnu@126.com (Z.F.); lingjun0217@163.com (J.L.); 2 Dongguan Institute of Jinan University, Dongguan 535000, China

**Keywords:** limbal stem cells, cryopreservation, inflammatory factor induction, cryogenic induction, primary cell culture

## Abstract

**Highlights:**

**What are the main findings?**

- Long-term cryopreserved limbal tissues retain viable LSCs with robust stem cell characteristics.
- A combined cryopreservation and IL-1β preconditioning strategy efficiently enriches LSCs and improves culture outcomes.

**What are the implications of the main findings?**

- Introduces a selection-based strategy for stem cell enrichment without complex sorting methods.
- Provides a practical approach for efficient LSC preparation prior to transplantation.

**Abstract:**

Limbal stem cell (LSC) transplantation is an important treatment for limbal stem cell deficiency (LSCD), but low efficacy in maintaining LSC stemness during in vitro expansion greatly affects its wider application. The primary contributing factors include a low proportion of stem cells and the lack of a stable, supportive microenvironment over prolonged culture. Rabbit corneal tissues preserved under deep cryogenic conditions for more than six months retain viable limbal stem cells (LSCs), and primary LSCs isolated from these tissues exhibit robust stem cell characteristics. It is noteworthy that the NLRP3/Caspase-1/IL-1β signaling axis was activated in corneal epithelial cells, and outer limbal layers preserved for one or two years. Based on these findings, a combined strategy integrating deep cryopreservation with IL-1β induction was established for the processing of limbal tissues. The combined cryogenic and IL-1β preconditioning yielded primary LSCs with maintained p63^+^ cell proportions, a reduction in K3^+^ differentiated cells from approximately 80% to 60%, and a 6.25-fold increase in colony-forming efficiency. In addition, an increased proportion of cells in the G2/M phase and enhanced proliferative capacity were observed. The enriched LSC population also exhibited improved stratified epithelial reconstruction potential. These findings identify an effective strategy for preserving and enriching LSCs from limbal tissue, providing a practical and efficient approach for LSC preparation prior to transplantation. Further in vivo studies will be important to validate the functional performance of these cells in ocular surface reconstruction.

## 1. Introduction

Vision is the primary sensory modality in humans, and maintaining a healthy ocular surface with an intact cornea and conjunctiva is crucial for optimal visual function. Corneal injuries such as chemical/thermal burns, infections, or iatrogenic damage can cause partial or complete limbal stem cell deficiency (LSCD), leading to corneal neovascularization, conjunctivalization, and potentially severe vision loss [1]. LSCs, located in the palisades of Vogt at the corneal epithelial basal layer, drive corneal epithelial renewal by migrating centripetally to replenish differentiated corneal epithelial cells, maintaining epithelial homeostasis [2]. To date, LSC transplantation has been widely recognized as a promising therapeutic approach for LSCD. Numerous researchers have devoted significant efforts to optimizing various culture techniques to enhance the stemness of transplanted cells and improve transplantation success rates.

In the clinic, donor corneas are usually preserved 1–2 weeks (short-term) in commercial storage media at 4–8 °C or in liquid nitrogen for a long-period [3,4,5]. After the transplantation of the central cornea, the limbal tissues of the donor corneas become potentially valuable resources for LSC cultivation. The feasibility of LSCs’ isolation from cryopreserved corneal tissue has been reported, but low temperature preservation could cause cell apoptosis and pyroptosis during direct cell freezing [6,7,8]. Reactive oxygen species (ROS) accumulation [9], ice crystal formation [10], and ionic imbalance [11] were assumed as the main factors. However, increasing studies have supported that applying proper negative stimulation to stem cells could enhance their bioactivities for regeneration. Therefore, further investigation into the cryodamage mechanisms affecting limbal tissues and LSCs may provide new insights for optimizing LSC cultivation.

Upon corneal epithelial injury, quiescent LSCs residing in the limbus would receive complex signals from the damaged site and rapidly exit their quiescent state under the coordinated regulation of inflammatory factors, growth factors, and niche microenvironment cues, initiating a series of regenerative responses [12]. Moreover, recent studies employing single-cell transcriptomics and quantitative lineage tracing have further confirmed the necessity of “appropriate inflammatory signaling” for LSC activation [13,14]. Our previous study developed a pretreatment method with collagenase IV and tumor necrosis factor-alpha (TNF-α) to simulate the epithelium injury, which significantly accelerated the outgrowth of primary LSCs while enhancing their stemness and viability [15].

In contrast to the corneal stroma, the limbal niche contains not only abundant growth factors but also various extracellular matrix (ECM) components enriched with vitronectin, fibronectin, laminin, tenascin, and Wnt ligands. These components collectively support the maintenance of stem cell phenotypes in LSCs under stress or injury conditions. Furthermore, the quiescent state of LSCs—characterized by slow cycling and low metabolic activity—provides inherent protection against external damage, so using cryogenic conditions may induce a more gradual and ordered injury to the corneal epithelium. Therefore, this study aims to investigate the stem cell characteristics, proliferative potential, and underlying regulatory mechanisms of primary LSCs derived from limbal tissues following long-term cryogenic preservation. A new pretreatment method for limbus tissue to acquire favorable LSCs would be established based on the discovered mechanism.

## 2. Materials and Methods

### 2.1. Preparations of Rabbit Limbus

The animal experiment procedures were conducted according to the ethical guidelines of the National Guide for the Care and Use of Laboratory Animals and approved by the Jinan University Animal Care and Use Committee (Guangzhou, China; approved code: 20240307-02). Following enucleation by orbital dissection, harvested rabbit eyeballs were immersed in 4 °C pre-cooled PBS (LEAGENE, Beijing, China) containing 1% penicillin–streptomycin (Gibco, Grand Island, NY, USA) for sterilization and removal of extraocular tissues. After meticulous removal of intraocular contents and iris tissue, corneoscleral rims were prepared by trimming excess sclera while preserving the limbal region. For cryopreservation, intact corneal tissues were equilibrated in specialized cryomedium (70%DMEM/F12 (Gibco, Grand Island, NY, USA), 30% fetal bovine serum (FengMingZhiYu, Dongguan, China), 10% Dimethyl sulfoxide (Solarbio, Beijing, China)) and subjected to controlled-rate freezing before long-term storage in liquid nitrogen. For the preconditioning group, the cryomedium was added with 10 ng/mL IL-1β, and the cornea tissues were stored in liquid nitrogen (−196 °C) for 7 days.

### 2.2. LSC Primary Culture

For primary culture, cryopreserved specimens were thawed and processed through PBS washing, followed by 3 mm limbal biopsy collection using a corneal trephine. Tissue explants were briefly coated with fetal bovine serum and plated epithelial-side-up in culture dishes with keratinocyte culture medium, maintaining standard culture conditions (37 °C, 5% CO_2_) with medium renewal every 48 h to monitor limbal stem cell outgrowth.

### 2.3. Limbal Stem Cells Quantifications

The expression levels of stem cell-related markers p63 (Abcam, Cambridge, UK), differentiation marker K3 (Abcam, Cambridge, UK), and proliferation marker Ki67 (Abcam, Cambridge, UK) were assessed using flow cytometry and immunofluorescence staining. Following overnight incubation with primary antibodies, samples were treated with species-matched secondary antibodies: Alexa Fluor 488-conjugated goat anti-mouse IgG (Abcam, Cambridge, UK) or goat anti-rabbit IgG (Abcam, Cambridge, UK). Negative controls were included for specificity validation. Nuclei were counterstained with Hoechst 33328 (Invitrogen, Carlsbad, CA, USA) for immunofluorescence imaging.

### 2.4. Cellular Proliferation Evaluation

Cellular proliferation was evaluated at 72 h using CCK-8 assays, while apoptosis rates were quantified with the Alexa Fluor^®^ 488 Annexin-V/Dead Cell Apoptosis Kit (Invitrogen, Carlsbad, CA, USA) according to manufacturer protocols. Flow cytometric analysis (BD FACSCanto, BD Biosciences, San Jose, CA, USA) was employed for both cell cycle profiling and marker quantification, ensuring standardized data acquisition and analysis.

### 2.5. H&E Staining

For histological evaluation, corneal tissues were fixed in 4% paraformaldehyde for 24 h, dehydrated through a graded ethanol and xylene series, and embedded in paraffin. Sections (5 μm thickness) were cut and mounted on slides, followed by rehydration through xylene and descending ethanol gradients. Hematoxylin and eosin (H&E) staining was performed for general morphological assessment.

### 2.6. Immunofluorescence Staining

For immunofluorescence analysis, antigen retrieval was conducted using citrate buffer. Tissue sections were incubated overnight at 4 °C with primary antibodies against p63, K3, NLRP3, IL-1β, and Caspase-1 (Abcam, Cambridge, UK). The following day, slides were incubated with appropriate secondary antibodies in the dark, followed by nuclear counterstaining with Hoechst 33328.

### 2.7. Real-Time Quantitative PCR

The TRIzol reagent (Invitrogen, Carlsbad, CA, USA) was used to extract total RNA. As directed by the manufacturer, cDNA synthesis was carried out using cDNA Reverse Transcription Kits (Servicebio, Wuhan, China). qRT-PCRs using the SYBR Green reagent (Servicbio, Wuhan, China) were performed using a Mini Cycler PCR device. Relative expression was calculated using 2^−ΔΔCT^ after gene expression was normalized to GAPDH mRNA. Table 1 contains the primer sequences for the genes that were detected. The primers were synthesized by GenScript Biotech Corporation (Nanjing, China).

### 2.8. RNA-Seq Analysis

According to the manufacturer’s protocol, a total RNA Kit I (Omega, Norcross, GA, USA) was used to extract sample RNA. Sequencing was performed on the Illumina platform. The raw RNA-seq reads were aligned by hisat2 (v. 2.1.0). Gene expression was calculated by htseq-count (v.0.11.2). Significantly differentially expressed genes (DEGs) among the fresh group and preconditioned group were evaluated using DESeq2 (1.22.2), and genes with a |log_2_FC|  ≥  1 and an adjusted *p* value ≤ 0.05 were selected for further analysis. Gene Ontology (GO), Kyoto Encyclopedia of Genes and Genomes (KEGG) enrichment analysis, and gene set enrichment analysis (GSEA) were conducted.

### 2.9. Statistical Analysis

All analyses were performed using GraphPad Prism 10.4.1 (GraphPad Software Inc., San Diego, CA, USA). A two-tailed Student’s *t*-test was used for comparisons of two groups. For multiple groups, one- or two-way ANOVA was used to compare. The number of samples in each experimental group was more than or equal to three (*n*  ≥  3). All quantitative results were expressed as the mean  ±  standard deviation, and differences with a *p* value < 0.05 were considered statistically significant.

## 3. Results

### 3.1. Long-Term Cryogenic Preservation in Liquid Nitrogen Maintains Stem Cell Phenotype and Viability in Rabbit Limbus

**Figure 1:**
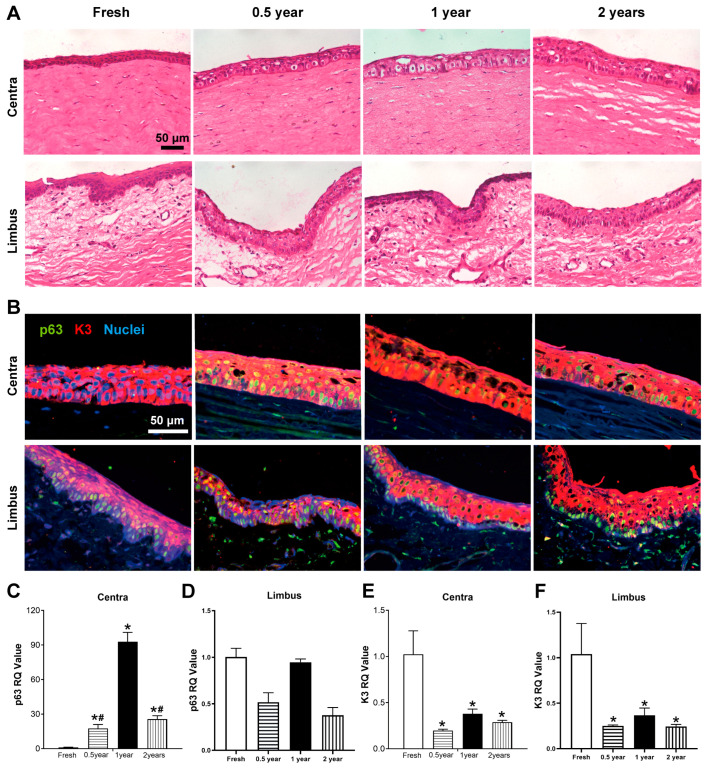
Structural and molecular characterization of rabbit corneas after different cryopreservation periods. (**A**) H&E staining of central cornea and limbus. (**B**) Immunofluorescence of p63 (stem cell marker, green) and K3 (differentiation marker, red) in corneal epithelium. (**C**–**F**) qPCR analysis of p63 and K3 mRNA levels in central cornea (**C**,**E**) and limbus (**D**,**F**). * *p* < 0.05 vs. Fresh; # *p* < 0.05 vs. 1-year group.

Histological examination by H&E staining demonstrated that the central corneal epithelium in fresh samples exhibited tightly arranged cells with intact cellular architecture. In contrast, rabbit corneas cryopreserved for 0.5, 1, and 2 years showed enlarged intercellular spaces and cytoplasmic vacuolization in the central corneal epithelium. The limbal region of fresh corneas displayed densely packed cells in both epithelial and basal layers. All three cryopreserved groups retained substantial epithelial cells in both limbal and central corneal regions, with the limbal basal layer maintaining numerous closely arranged cells (Figure 1A). Immunofluorescence staining for p63 and K3 revealed that both fresh and cryopreserved corneas showed positive K3 expression in central corneal epithelial cells, with only a minority of cells expressing p63. Compared with the fresh group, the cryopreserved groups expressed higher p63 in the corneal area. In the limbal region, p63^+^ cells were predominantly localized in the basal layer of both fresh and cryopreserved corneas, while K3 expression was concentrated in the superficial layers with minimal expression in the limbal basal region (Figure 1B). qRT-PCR analysis indicated that p63 transcript levels in the central cornea were elevated in all three cryopreserved groups compared to fresh controls (Figure 1C), whereas no significant differences were observed in p63 expression between cryopreserved and fresh limbal tissues (Figure 1D). Additionally, K3 transcript levels were reduced in both central corneal and limbal tissues of cryopreserved groups relative to fresh samples (Figure 1E,F).

### 3.2. Using Liquid Nitrogen Cryopreservation Limbus for LSC Primary Culture

**Figure 2:**
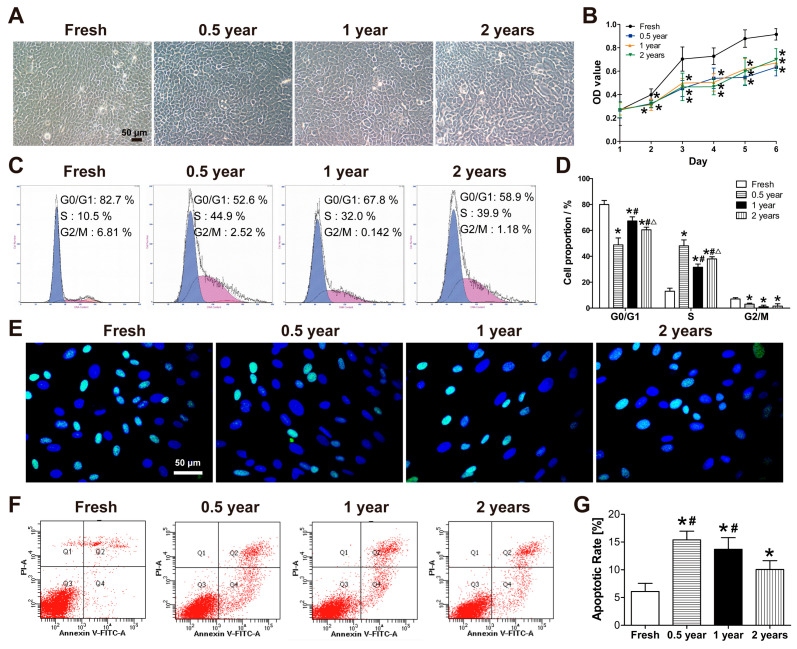
Proliferative activity of primary LSCs isolated from fresh and cryopreserved rabbit limbus. (**A**) Primary LSCs morphology under the bright field microscope. (**B**) Cell proliferation curves measured by CCK-8 assay. (**C**) Cell cycle analysis (G0/G1 phase by blue, S phase by purple and G2/M by orange ) by flow cytometry. (**D**) Statistical results of cell cycle distribution (G1, S, and G2 phases; *n* = 3). (**E**) Immunofluorescence staining of Ki67 (green) and nuclei(blue). (**F**) Apoptosis rate analysis by flow cytometry. (**G**) Statistical results of apoptosis rates (*n* = 3). * *p* < 0.05 vs. Fresh; # *p* < 0.05 vs. 0.5 year; △ *p* < 0.05 vs. 1 year (**D**); * *p* < 0.05 vs. Fresh; # *p* < 0.05 vs. 2 years.

**Figure 3:**
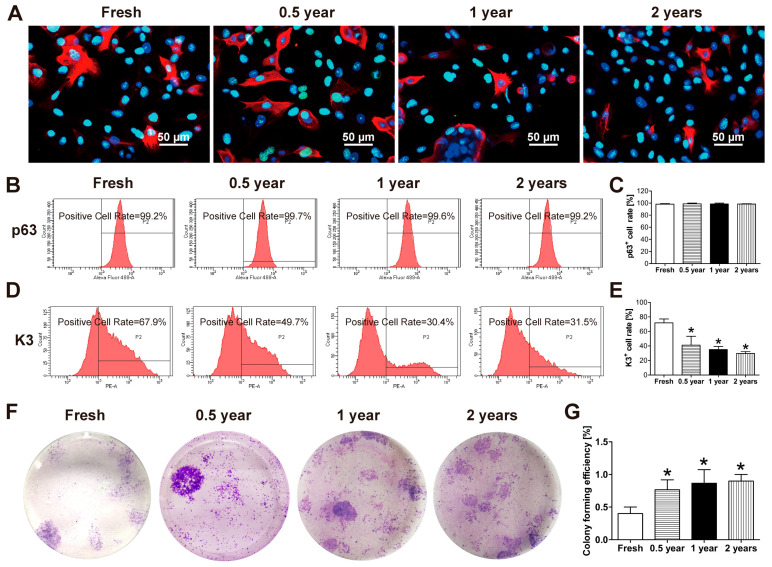
Stem cell characteristics of primary LSCs from fresh and cryopreserved rabbit corneas. (**A**) Immunofluorescence staining of p63 (green), K3 (red), and nuclei (blue). (**B**,**C**) Flow cytometric analysis and quantification of p63^+^ cells. (**D**,**E**) Flow cytometric analysis and quantification of K3^+^ cells. (**F**) Representative images of colony formation assay with Giemsa staining. (**G**) Quantification of colony-forming efficiency. * *p* < 0.05 vs. Fresh.

Primary culture of LSCs from rabbit limbus was performed using the explant adherence method. Fresh corneas showed cell outgrowth by day 2–3, whereas corneas cryopreserved for 0.5, 1, and 2 years showed delayed outgrowth until day 4–5. The morphology of primary LSCs between fresh and cryopreserved groups was similar, with both exhibiting polygonal, cobblestone-like arrangements (Figure 2A). Growth curve analysis revealed that LSCs derived from 0.5-, 1-, and 2-year cryopreserved corneas all exhibited reduced proliferative rates compared to the fresh control group, and no significant differences were observed among the cryopreservation groups (Figure 2B). Cell cycle analysis demonstrated higher proportions of G0/G1 phase and G2 phase cells in the fresh group compared to the cryopreserved groups. Conversely, the S-phase cell population was lower in the fresh group than in all cryopreserved groups (Figure 2C,D). Immunofluorescence staining confirmed Ki67 expression in both fresh and cryopreserved groups (Figure 2E). Apoptosis assays revealed the lowest apoptosis rate in fresh LSCs, while the highest rates were observed in the 0.5- and 1-year cryopreserved groups (Figure 2F,G). In addition, immunofluorescence staining and flow cytometric analysis of LSC markers showed that the proportion of p63^+^ cells in primary LSCs derived from cryopreserved limbus was not significantly different from that in the fresh group (Figure 3B,C), whereas the proportion of K3^+^ cells was markedly reduced (Figure 3D,E). Moreover, the colony-forming efficiency of primary LSCs from all three cryopreserved groups was significantly higher than that of the fresh group (Figure 3F,G).

### 3.3. The NLRP3/Caspase-1/IL-1β Signaling Pathway Participates in the Modulation of Cryopreservation-Induced Epithelial Cell Damage

**Figure 4:**
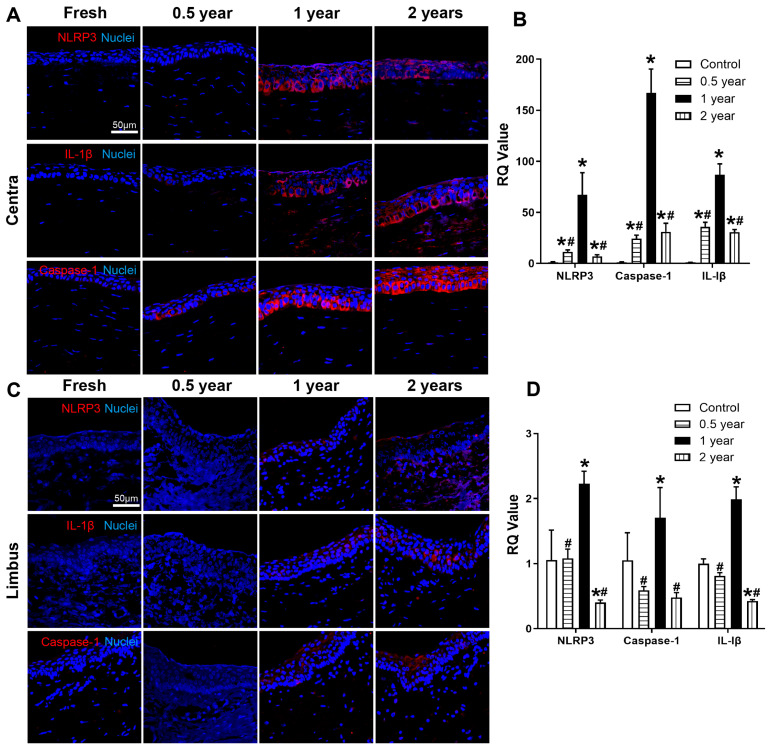
Expression of NLRP3, Caspase-1, and IL-1β in fresh and cryopreserved corneas. (**A**) Immunofluorescence staining and (**B**) mRNA expression of NLRP3, Caspase-1, and IL-1β in the central cornea of fresh and corneas cryopreserved for 0.5, 1, and 2 years. (**C**) Immunofluorescence staining and (**D**) mRNA expression of the indicated markers in the limbal cornea. *n* = 3. * *p* < 0.05 vs. fresh; # *p* < 0.05 vs. 1 year.

Immunofluorescence staining of corneal sections revealed distinct patterns of NLRP3, Caspase-1, and IL-1β expression in fresh versus cryopreserved tissues. Fresh corneal tissues showed no detectable positive staining for any of these markers. In contrast, cryopreserved corneas exhibited progressively increased numbers and staining intensity of NLRP3+, Caspase-1+, and IL-1β+ cells in the central corneal epithelium with longer storage durations (Figure 4A). qPCR analysis confirmed these findings at the transcriptional level, with fresh corneas demonstrating significantly lower expression of NLRP3, Caspase-1, and IL-1β mRNA compared to all cryopreserved groups. Among cryopreserved samples, the 1-year group showed the highest transcript levels of all three markers in the central cornea, while the 0.5- and 2-year groups exhibited intermediate expression levels (Figure 4B). The limbal region displayed markedly different expression patterns. Both fresh and 0.5-year cryopreserved limbal tissues were negative for all three markers by immunofluorescence. Only the superficial layers of 1- and 2-year cryopreserved limbal tissues showed positive staining, while the limbal basal layer remained consistently negative across all groups (Figure 4C). Transcriptional analysis revealed that 1-year cryopreserved limbal tissues had elevated NLRP3, Caspase-1, and IL-1β mRNA levels compared to fresh controls, whereas the 2-year group showed significantly reduced expression. No significant differences were observed between fresh and 0.5-year cryopreserved limbal tissues (Figure 4D).

### 3.4. Combined Cryopreservation and Inflammatory Preconditioning Enable Selection of High-Viability Primary Limbal Stem Cells

**Figure 5:**
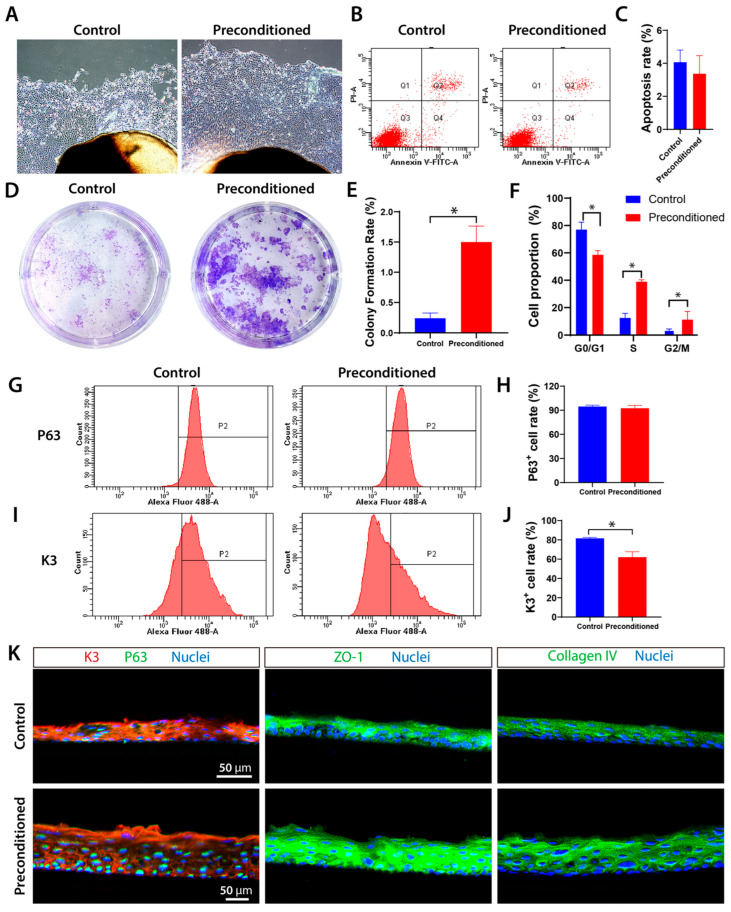
Biological characterization of rabbit primary LSCs following combined cryogenic treatment and IL-1β inflammatory induction. (**A**) Morphology of primary LSCs migrating from fresh corneas and IL-1β-treated cryopreserved limbal tissues at day 3. (**B**,**C**) Analysis of apoptosis rates. (**D**) Representative images of colony-forming assay with Giemsa staining, and (**E**) quantification of colony-forming efficiency (* *p* < 0.05 vs. Control). (**F**) Cell cycle analysis (* *p* < 0.05 vs. Control). (**G**,**H**) Flow cytometric analysis and quantification of p63^+^ cells. (**I**,**J**) Flow cytometric analysis and quantification of K3^+^ cells (* *p* < 0.05 vs. Control). (**K**) Construction of multilayered LSC sheets in vitro and immunofluorescence staining results.

Using fresh limbal tissue as a control, primary LSCs were isolated from cryopreserved and inflammation-induced limbal tissues (preconditioned group) to evaluate their stem cell properties. Both groups yielded primary LSCs with polygonal, cobblestone-like morphology, showing no apparent morphological differences. Compared to the control group, the preconditioned group exhibited significantly greater LSC outgrowth by day 3, while apoptosis assays revealed no significant differences between groups (Figure 5A–C). The preconditioned group demonstrated superior stem cell functionality, as evidenced by significantly higher colony-forming efficiency (Figure 5D,E) and altered cell cycle distribution—with reduced G0/G1 phase populations and increased S/G2-M phase fractions compared to controls (Figure 5F). Flow cytometry analysis showed comparable p63^+^ cell proportions between groups, but significantly fewer K3^+^ cells in the preconditioned group (Figure 5G–J). In vitro stratified culture revealed enhanced tissue-forming capacity in the preconditioned group, generating 5–6 layered sheets versus 3–4 layers in controls. Immunofluorescence demonstrated distinct differentiation patterns: control sheets showed uniform K3 expression with weak basal p63, while pretreatment sheets exhibited stronger superficial K3 and intense basal p63 expression. Both groups maintained robust expression of tight junction protein ZO-1 and collagen IV throughout the stratified constructs (Figure 5K).

### 3.5. Inflammatory Preconditioning Enhances the Activity of Primary LSCs by Upregulating Extracellular-Related Gene Expression

**Figure 6:**
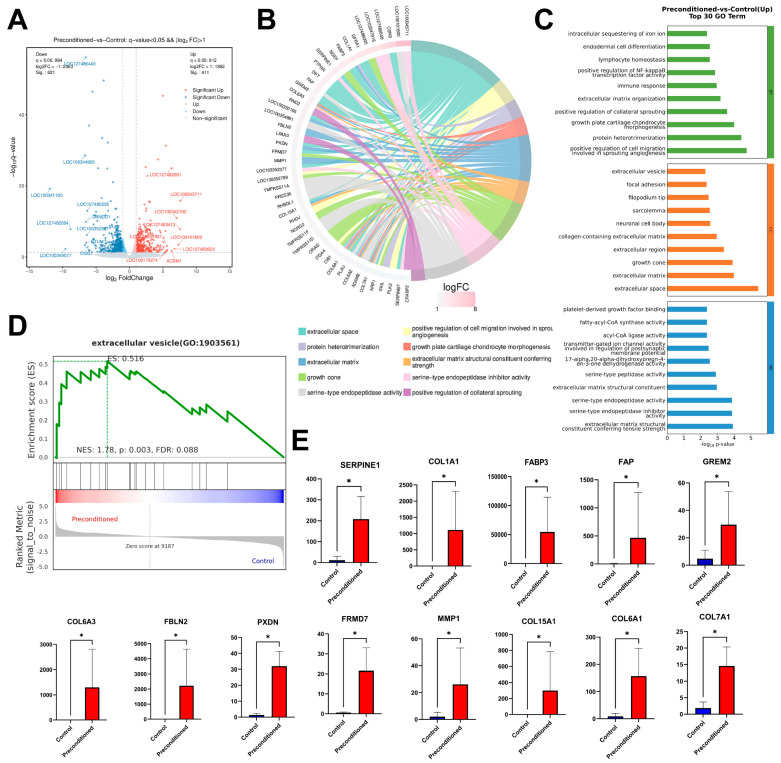
Transcriptomic analysis identifies key upregulated genes. (**A**) Volcano plot of differentially expressed genes (DEGs). (**B**) Chord plot of GO enrichment analysis for upregulated DEGs. (**C**) Top 30 significantly enriched GO terms for upregulated DEGs. (**D**) Gene Set Enrichment Analysis (GSEA) reveals upregulation of extracellular vesicle-related pathways. (**E**) qPCR validation of selected upregulated genes (* *p* < 0.05 vs. Control).

Based on the transcriptomic analysis, 1032 genes were found to be significantly different between the fresh group and the preconditioned group (Figure 6A). According to the GO enrichment analysis, many of the significantly upregulated genes in the preconditioned group were enriched in extracellular-related pathways, including the extracellular matrix, extracellular space, and extracellular vesicles (Figure 6B,C). Among the significantly upregulated genes, we selected SERPINRE1, COL1A1, FABP3, FAP, GREM2, COL6A3, FBLN2, PXDN, FRMD7, MMP1, COL15A1, COL6A1, and COL7A1 for qPCR validation (Figure 6E), and the results were consistent with the transcriptomic analysis. However, in the GSEA, only the extracellular vesicle pathway showed significant upregulation (Figure 6D).

## 4. Discussion

LSCs are essential for maintaining ocular surface homeostasis and function, and their transplantation remains an effective strategy for ocular surface reconstruction. However, the clinical application of LSCs has been hindered by difficulties in maintaining stemness during expansion, the low proportion of stem cells, and the reduced viability of residual corneal tissues and LSCs after long-term storage. In this study, we established a strategy that combines deep cryogenic preservation with inflammatory factor induction to induce and enrich LSCs within limbal tissues. This approach not only preserved stem cell characteristics during cryopreservation but also yielded a higher proportion of LSCs with robust activity. These findings provide a promising method for generating high-quality LSCs and may offer a reliable cell source for transplantation-based therapies of ocular surface disorders.

Previous studies have reported that cryogenic conditions can induce DNA damage [16], cell detachment, and apoptosis in corneal epithelial cells [3]. Consistent with these findings, our results showed partial epithelial cell detachment in corneal tissues after long-term cryogenic storage in liquid nitrogen. Nevertheless, LSCs located in the limbus retained their stem cell phenotype and proliferative capacity. Even after up to two years of cryopreservation, a considerable proportion of p63^+^ LSCs remained in the limbal tissue, suggesting that cryopreserved corneas may still serve as a viable source of LSCs. Activation of stem cells is a key process in tissue regeneration. During corneal wound healing, epithelial cells at the injury site first release inflammatory cytokines such as IL-1 and TNF-α, followed by apoptosis, after which growth factors, including EGF, TGF-β1, VEGF, and HGF, are produced, ultimately leading to epithelial cell migration, proliferation, and wound closure [12,17]. Quiescent LSCs located in the limbal niche rapidly switch to an activated state upon receiving specific signals, such as inflammatory cytokines and growth factors, thereby initiating proliferation while maintaining their stem cell phenotype [18]. An increased p63 signal was observed in the central cornea following cryopreservation. This change may be associated with stress- and inflammation-induced epithelial activation [19]. However, the precise cellular origin and regulatory mechanisms underlying this phenomenon were not specifically addressed and warrant further investigation. The immunofluorescence results demonstrated that long-term cryogenic storage activated the NLRP3/Caspase-1/IL-1β pathway in corneal epithelial cells, particularly in central corneal epithelium and the superficial layers of limbal epithelium (Figure 4). In contrast, LSCs residing in the basal limbal layer did not express NLRP3, Caspase-1, or IL-1β (Figure 4C). This may be attributed to the unique folded structure of the limbal niche, which could mitigate direct cryogenic stress and prevent inflammasome activation, thereby protecting LSCs from pyroptosis. Together, these findings highlight the differential impact of deep cryopreservation on highly differentiated corneal epithelial cells versus limbal stem cells, which provides theoretical support for strategies aimed at activating LSCs and expanding highly viable LSCs in vitro.

To evaluate the feasibility of obtaining LSCs with robust stem cell activity from long-term cryopreserved corneal tissues, we performed primary isolation and biological assessment of limbal tissues stored in liquid nitrogen for up to two years. In vitro, fresh LSCs showed higher proliferative activity compared with cryopreserved LSCs (Figure 2B). Cell cycle analysis revealed that most cryopreserved LSCs were arrested in the S phase (Figure 2C,D). The cryopreserved LSCs exhibited a higher rate of apoptosis (Figure 2F,G). Notably, LSCs isolated from cryopreserved corneas in our study demonstrated a higher colony-forming efficiency (Figure 3F), while maintaining a comparable proportion of p63^+^ cells to fresh LSCs and exhibiting a significantly lower proportion of K3^+^ differentiated cells (Figure 3B,D). These results suggest that long-term cryopreservation may trigger pyroptosis-associated signaling in limbal niches, thereby activating quiescent LSCs, initiating proliferative programs. In vivo, LSCs are known to be slow-cycling, whereas in culture they become activated and proliferative, with rapid expansion largely driven by transient amplifying cells [20]. The cryopreserved group exhibited a higher apoptotic rate while showing enhanced cloning-forming capacity and a reduced proportion of K3^+^ differentiated cells, suggesting that cryopreservation imposes greater damage on differentiated or transient amplifying cells, thereby selectively enriching for less differentiated LSCs. This may represent a purification mechanism that eliminates terminally differentiated cells while preserving LSCs with superior stem cell properties. However, long-term cryopreservation may still cause damage to the limbal niche and induce DNA damage in resident cells, driving cells into a repair-associated S phase and resulting in an increased proportion of S-phase cells in the cryopreserved group. Therefore, our aim is to establish a strategy that preferentially promotes apoptosis of differentiated cells while minimizing cellular damage, thereby enabling the selection and enrichment of p63^+^ LSCs with higher cloning-forming efficiency and stem cell characteristics.

To preserve and enrich LSCs with superior stem cell characteristics within limbal tissues, this study employed a combined deep cryogenic and IL-1β–induced preconditioning strategy. The results showed that limbal tissues pretreated with IL-1β-supplemented cryopreservation medium for one week yielded more primary LSCs within the same time compared to the fresh control group (Figure 5A). The LSCs from the preconditioned group have significantly higher colony-forming capacity (Figure 5D), indicating that the preconditioned group cells have enhanced self-renewal potential and proliferative competence. Also, flow cytometry analysis showed that the preconditioned group exhibited a similar proportion of p63^+^ cells (LSCs) but fewer K3^+^ differentiated cells (Figure 5G–J), suggesting that the preconditioning favors the enrichment of stem cells. Furthermore, the multilayered LSC sheets formed by the preconditioned group have more p63^+^ cells and high expression of ZO-1 and collagen IV (Figure 5K). The enhanced colony-forming capacity and stratification capacity highlight its promise for future applications in LSC transplantation and tissue-engineered corneal constructs.

In addition, RNA-seq analysis was performed to investigate the potential mechanisms by which cryogenic and inflammatory factor induction enhances LSCs’ activity. In the GO enrichment analysis, multiple significantly upregulated genes were found to be associated with the extracellular space and extracellular matrix. However, the proteins regulated by these genes exert distinct effects on the ECM. The upregulation of COL1A1, COL6A3, COL15A1, COL6A1, COL7A1, FABP3, FBLN2, GREM2, and PXDN can promote ECM production and increase matrix stiffness [21,22,23,24,25]. This is likely a biological response to inflammatory induction, in which cells maintain stemness by strengthening the niche through more ECM deposition. Meanwhile, SERPINE1, FAP, FRMD7, and MMP1 can alter ECM composition through degradation or remodeling, and promote cell migration [26,27,28]. This may reflect the activation of LSCs under inflammatory stimulation, enabling them to migrate more rapidly toward the site of injury or out of the limbal niche under in vitro culture conditions. These results indicate that the regulation of ECM by cryopreservation and inflammatory induction is likely dynamic rather than unidirectional. This dynamic regulation enables the limbal tissue to yield a higher purity of LSCs with faster migration capacity after induction. In the GSEA based on the whole transcriptome, the extracellular vesicle pathway was significantly enriched, suggesting enhanced extracellular vesicle (EV)-mediated intercellular communication under these conditions. Evidence indicated that EVs play important roles in modulating ECM dynamics and microenvironmental remodeling, particularly under stress or inflammatory conditions [29], where EV secretion and cargo composition are altered. Through the transfer of bioactive molecules, EVs can influence ECM turnover, cellular plasticity, and tissue homeostasis [30]. These observations raise the possibility that the dynamic regulation of ECM may be partially mediated by EV-dependent signaling. However, this hypothesis remains speculative and requires further experimental validation.

## 5. Conclusions

Corneal tissues preserved under cryogenic conditions retained viable LSCs, and the isolated primary LSCs exhibited strong stemness and colony-forming efficiency. By combining cryogenic preservation with IL-1β induction, we established an optimized approach that yields LSCs with a higher stem cell proportion and enhanced proliferative potential. This strategy provides an effective means to preserve and enrich LSCs within limbal tissues, offering an effective safeguard for LSC transplantation and other related applications.

## Figures and Tables

**Table 1:** Primers for qPCR.

SERPINE1 FWD	TCGAGGTGAATGAGAGTGGC
SERPINE1 REV	AAAGGATGGTACCTGTGGGG
COL1A1 FWD	AGAACGGAGATGACGGAGAAG
COL1A1 REV	CTTGGCACCATCCAAACCAC
FABP3 FWD	ATGACAGGAAGGTCAAGTCCC
FABP3 REV	TTTCTCGTAAGTGCGTGTGC
FAP FWD	GATGAGGGGGTCGCATACAG
FAP REV	TAGTTGGAAGCCGAAGCCAG
GREM2 FWD	TGCGGGTTTAGGGGATGAAG
GREM2 REV	CACACACTTCAGTCGCTTGC
COL6A3 FWD	TCAACGCCCTGCAGATCAAC
COL6A3 REV	TCCAGTTGCCGAATCCACAG
FBLN2 FWD	CGTCTCCTGTGAGGACCAAG
FBLN2 REV	GTGCGCGTTGAGGATAAAGC
PXDN FWD	TGACAGGCAAGCGTTTAAGG
PXDN REV	CAGGTGTGTGATCCGGTTGT
FRMD7 FWD	CTATTGATTGTCAGCACTCGGC
FRMD7 REV	GGGGGCTTTCTCCAGGAATTT
MMP1 FWD	GGCTCAGTTCGTCCTCACTC
MMP1 REV	TGGTGAATGTCAGGGGTGTG
COL15A1 FWD	CTTGCCTTCCATCCCAACCT
COL15A1 REV	ATCAAGGAAGGACCCATCGC
COL6A1 FWD	CAAAGGCACCTACACCGACT
COL6A1 REV	CCCATCGGTCACCACAATCA
COL7A1 FWD	TCACGAACCAGTCCTCATGC
COL7A1 REV	ACTGCCGAAGTCAGGTCTTG

## Data Availability

The RNA-seq data from limbal stem cells used in this study are available in the GEO database under accession code “GSE329824”. Any additional requests for information can be directed to, and will be fulfilled by, the corresponding authors.
